# High negative predictive value of 68Ga PSMA PET-CT for local lymph node metastases in high risk primary prostate cancer with histopathological correlation

**DOI:** 10.1186/s40644-019-0273-x

**Published:** 2019-12-11

**Authors:** Labib Ataur Rahman, Damacent Rutagengwa, Peter Lin, Michael Lin, June Yap, Ken Lai, Pascal Mancuso, Prem Rathore, Kayvan Haghighi, Paul Gassner, Lee Hao Wong, Nestor Lalak

**Affiliations:** 1 Department of Nuclear Medicine and PET, Liverpool Hospital, Sydney, Australia; 2 South Western Sydney Clinical School, University of New South Wales, Sydney, Australia; 3 Department of Urology, Campbelltown Hospital, Sydney, Australia; 4 School of Medicine, Western Sydney University, Sydney, Australia; 5 Department of Urology, Liverpool Hospital, Sydney, Australia

**Keywords:** Prostate Cancer, PET, PSMA - prostate specific membrane antigen, Primary staging, Lymph node, Prostatectomy

## Abstract

**Background:**

Current guidelines highlight the importance of accurate staging in the management and prognostication of high risk primary prostate cancer. Conventional radiologic imaging techniques are insufficient to reliably detect lymph node metastases in prostate cancer. Despite promising results, there is limited published data on the diagnostic accuracy of PSMA PET-CT to assess local nodal metastases prior to radical prostatectomy.

This study aims to assess the diagnostic efficacy of 68Ga PSMA PET-CT in local lymph node staging of high risk primary prostate cancer when compared to histopathological findings following radical prostatectomy with pelvic lymph node dissection.

**Methods:**

We retrospectively analysed consecutive patients with high risk primary prostate cancer referred by urologists for primary staging PSMA PET-CT using a 68Ga-labeled PSMA ligand, Glu-NH-CO-NHLys-(Ahx)-[HBEDD-CC], from October 2015 to October 2017. The scans of patients who underwent radical prostatectomy with pelvic lymph node dissection were interpreted by the consensus reading of two experienced nuclear medicine physicians blinded to clinical and histopathological data. The contemporaneous records of the referring urologists were retrospectively reviewed for noteworthy unexpected PET findings that altered their personal preference for surgical management.

**Results:**

Seventy-one patients were recruited and analysed. PSMA PET-CT showed findings compatible with local disease in 47 patients (66.2%), lymph node metastases in 10 patients (14.1%) and distant metastases in 14 patients (19.7%). Twenty-eight patients (twenty-seven of whom had local disease only) underwent surgery yielding 214 lymph nodes, all of which were negative on histopathological analysis. On a node-based analysis, 213 of 214 lymph nodes were accurately identified as negative for disease with a negative predictive value of 100%. 11 patients had unexpected PET findings contemporaneously documented by urologists to alter their preference for surgical management.

**Conclusions:**

PSMA PET-CT appears to have a high negative predictive value for local lymph node metastases in high risk primary prostate cancer when compared to histopathological findings following radical prostatectomy with pelvic lymph node dissection.

## Background

When employed in appropriately selected patients, radical prostatectomy (RP) with or without pelvic lymph node dissection (PLND) and/or additional multimodal therapy offer potential cure for prostate cancer (PCa). At present, controversy remains regarding how best to individualise treatment and select candidates for surgical therapy which often relies on risk-adapted nomograms. Current guidelines highlight the importance of accurate staging in the management and prognostication of high risk primary PCa [1].

Conventional imaging strategies for PCa rely on non-specific size criteria to define abnormal local lymph nodes despite 80% of lymph node metastases not meeting the standard 8 mm threshold^2^. As such, the gold standard for lymph node staging remains extended pelvic lymph node dissection. For practical reasons however, not all patients are candidates for or elect to undergo operative management. Furthermore, extended PLND can be associated with an overall complication rate of up to 20% [1].

Prostate specific membrane antigen (PSMA) Positron Emission Tomography-Computed Tomography (PET-CT) is emerging as a powerful new staging tool that is being rapidly adopted into clinical practice. PSMA is a type II transmembrane protein seen in the prostate which is expressed more strongly in the setting of cancer. Although 5–10% of PCas may not express PSMA, studies have demonstrated that 98% of lymph node metastases express PSMA [2, 3]. The use of PSMA PET-CT has been most widely studied in the setting of biochemical recurrence (BCR) of PCa. A systematic review found few high-quality studies investigating the use of PSMA PET-CT in the primary staging of high risk patients despite appearing to outperform conventional imaging modalities [4]. Furthermore, a recent study has shown that when used in the primary staging of intermediate and high risk PCas, management was altered in 21% of patients when compared with conventional imaging strategies [2, 5, 6]. To date, there have been several studies assessing the diagnostic performance of PSMA PET-CT in detecting lymph node metastases in the setting of primary prostate cancer with histopathological confirmation. The reported sensitivity has ranged from 33 to 84%, specificity from 84 to 100%, positive predictive value from 61.5–100% and negative predictive value from 69 to 98% [7–11].

Our aim was to assess the diagnostic efficacy of 68Ga PSMA PET-CT in the local lymph node staging of high risk primary prostate cancer when compared to histopathological findings after radical prostatectomy with pelvic lymph node dissection (RP-PLND).

## Methods

We conducted a retrospective review of consecutive patients from October 2015 to October 2017 with high risk PCa referred independently by urologists employed at our institution (Liverpool Hospital, Sydney, Australia) for primary staging PSMA PET-CT scans using a 68Ga-labeled PSMA ligand, Glu-NH-CO-NHLys-(Ahx)-[HBEDD-CC]. During the study period, referrals were typically made after conventional imaging had already been acquired and reviewed. Risk assessment was based on the European Association of Urology (EAU) risk groups for BCR of localised and locally advanced PCa since they are widely adopted in Australian clinical practice. High risk was defined as Prostate Specific Antigen (PSA) > 20 ng/mL or Gleason Score (GS) > 7 or TNM Stage cT2c or greater [1]. Patients being treated for a synchronous cancer were excluded. Patients who underwent RP-PLND either alone or as part of multimodal definitive therapy were selected for further analysis. The template for PLND was left to discretion of the surgeon as was the decision to perform the procedure with robotic assistance. Surgeons were not blinded to the results of the PSMA PET-CT scan. Treatment and follow-up were not necessarily performed at our institution. In order to highlight noteworthy cases, the contemporaneous records of the referring urologists were retrospectively reviewed for unexpected PET findings that were documented to alter their personal preference to offer or decline surgery. In October 2019, our local database was examined for available follow-up data on patients managed surgically within our health service.

All studies were acquired on a GE-Discovery-710 time-of-flight PET-CT Scanner. The target dose was 2 MBq/kg (dose range: 80-200 MBq). The PET studies were acquired in 3D mode for a total acquisition time of 1.5–2.5 min per bed position adjusted according to the patient weight, from midbrain to proximal femora at about 45–60 min post injection. Transmission scans and attenuation corrections were obtained by a 64-slice CT, using helical mode without the use of a contrast medium. CT images were acquired at 3.75 to 5 mm slice thickness and reconstructed to a transaxial matrix size of 512 × 512. The current (30–40 mAs) and voltage (120–140 kV) were varied according to the patient weight. The PET images were reconstructed using GE VUE Point FX (Time of Flight) algorithm into a 256 × 256 matrix size with a slice thickness of 3.75 to 4.0 mm. Patients were routinely administered 20 mg frusemide intravenously at the time of PSMA injection.

The scans of patients who underwent RP-PLND were interpreted by the consensus reading of two experienced nuclear medicine physicians blinded to prior clinical, imaging and histopathological data. Discrepant interpretations were resolved by a third experienced Nuclear Medicine Physician. Visual and quantitative analyses were performed on an Advantage Workstation using the PET-VCAR (Volume-Computer-Assisted-Reading) software.

For each scan, the Nuclear Medicine Physicians identified suspicious lesions visually using the criteria outlined by Fanti et al. [12]. They defined ‘anomalous uptake’ as any uptake higher than adjacent background at sites not expected to show physiologic uptake (including lacrimal and salivary glands, liver, spleen, small intestine, colon, kidneys and coeliac ganglia). ‘Pathologic uptake’ was defined as any anomalous uptake which is not better explained by another cause and is suggestive of prostate cancer.

The reference standard for this study was histopathological confirmation using a node-based analysis. Histopathological findings were reported at several different pathology centres. Data was collected from the available local electronic medical records and the private practices of the referring urologists. We performed a descriptive analysis of the data.

## Results

**Fig. 1:**
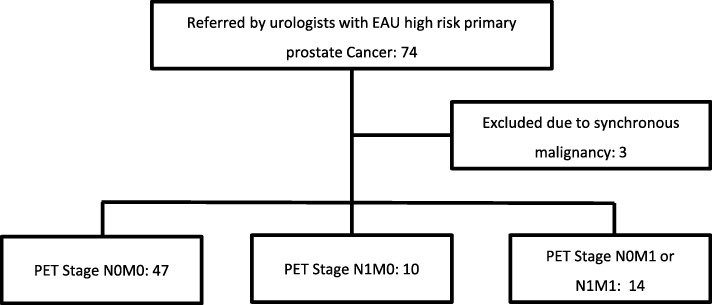
Flow chart of treatment for EAU high risk primary prostate cancer patients. RP-RPLND, Radical prostatectomy with pelvic lymph node dissection

Seventy-four patients were referred by urologists for PSMA PET-CT scans between October 2015 to October 2017 for primary staging of high-risk prostate cancer (Fig. 1). Three patients were excluded due to the presence of synchronous cancers. PSMA PET-CT showed findings compatible with local disease in 47 patients (66.2%), lymph node metastases in 10 patients (14.1%) and distant metastases in 14 patients (19.7%).

Twenty-eight patients underwent RP-PLND including twenty-seven patients with local disease only and one patient with local nodal disease in a single 4 mm right internal iliac lymph node with maximum Standardised Uptake Value of 4.1. Table 1 describes the baseline characteristics of the patients who underwent RP-PLND. A total of 214 lymph nodes were retrieved at surgery, all of which were negative on histopathological analysis. 213 of 214 lymph nodes were accurately identified as negative for disease using PSMA PET-CT with a negative predictive value of 100% using a node-based analysis. On a patient-based analysis, 27 of 28 patients had accurate lymph node staging using PSMA PET-CT.

**Table 1:** Baseline characteristics of twenty-eight patients who underwent RP-PLND

Median age in years at time of scan (range)	69 (46–82)	
Gleason Score: median (range)	8 (6–9)	
Prostate Specific Antigen (ng/mL): median (range)*	8 (3–51)	
TNM Stage Prior to PSMA PET-CT Scan (number of patients)	T1c	1
	T2	8
	T2c	8
	T3	6
	T3a	1
	T3b	1
	N1	1
	M1a	1
	M1b	1

*Data was not available for one patient

Table 2 summarises noteworthy cases in which unexpected PSMA PET-CT findings were contemporaneously documented to alter the individual urologist’s preference to offer or decline surgical management. This included 9 patients who were up-staged by the PET scan and 2 patients who were down-staged and subsequently underwent RP-PLND.

**Table 2:** Unexpected PSMA PET-CT findings contemporaneously documented to alter the referring urologist’s preference regarding surgical management

Case No.	Pre-PET TNM Stage	Urologist’s Pre-PET Preference To Offer Surgery	Post-PET TNM Stage	Unexpected PET Finding	Treatment Performed
1	M1a (para-aortic lymph nodes)	Decline	N0 M0	No lymph node PSMA uptake	RP-PLND
2	N0 M0	Offer	N1M1a	Non-regional lymph node metastases (extending above diaphragm)	Systemic therapy
3	T3M1b (lumbar spine and iliac bone)	Decline	N0 M0	No bony PSMA uptake	RP-PLND
4	T3 N0 M0	Offer	N1 M0	Presacral and internal iliac lymph node metastases	Whole pelvis radiotherapy and hormone therapy
5	T3aN0 M0	Offer	N0 M1	Solitary vertebral metastasis	Whole pelvis radiotherapy, hormone therapy, stereotactic radiotherapy to metastatic deposit
6	T3aN0 M0	Offer	N1 M0	Presacral and internal iliac lymph node metastases	Whole pelvis radiotherapy and hormone therapy
7	T2cN0 M0	Offer	N1M1b	Non-regional lymph node and bony metastases	Systemic therapy
8	T1 N0 M0	Offer	N1, possible M1b	Local lymph node metastases and possible iliac bone PSMA uptake	Whole pelvis radiotherapy and hormone therapy
9	T3 N1 M0	Offer	N1M1b	Non-regional lymph nodes and multiple bony metastases	Hormone therapy
10	T2cN0 M0	Offer	N1M1b	Non-regional lymph nodes and bony metastases	Hormone therapy
11	T3 N0 M0	Offer	N1M1a	Non-regional lymph node metastases	Hormone therapy

*RP-RPLND* Radical prostatectomy with pelvic lymph node dissection

By October 2019, the single patient who had nodal disease on the staging PSMA PET-CT scan prior to RP-PLND was the only patient to have had imaging-proven recurrence on a subsequent PET scan at our institution. BCR was seen at 8 months post-operatively and a repeat PET scan at 20 months (with PSA 0.9 ng/mL) showed multiple PSMA-avid pelvic lymph nodes, including in the right internal iliac region where lymph node metastases were seen on the initial staging scan.

Follow-up data was available in our local health service database for seventeen patients in October 2019. Mean duration of follow up was 22.8 months (range 6–37 months). There were four cases of BCR, including the aforementioned case, with a mean time to BCR of 11 months and a range of 8–18 months. There were also four cases of rising PSA treated with early salvage radiotherapy at low PSA levels (< 0.1 ng/mL). Mean time to rising PSA was 20.8 months (range 17–23 months).

## Discussion

We found that PSMA PET-CT had a high negative predictive value for primary staging of local lymph nodes in patients with high risk PCa compared to histopathological findings after RP-PLND. These results compared favourably to what has been previously reported in the literature [7–11]. Moreover, given that the estimated risk of positive lymph nodes in all high risk PCa patients treated with RP-PLND is 15–40%, a negative PSMA PET-CT scan appeared to select a subgroup that were less likely to have local lymph node involvement at surgery than existing risk nomograms would predict [1]. It is worth noting that due to the retrospective nature of our study, surgical lymph node dissection templates were not standardised prior to the study. With respect to the patient who underwent RP-PLND with nodal disease on the staging PSMA PET-CT scan, it is possible that the lymph node was not taken during PLND given that nodal metastases were seen in the same lymph node region on the post-operative PET scan.

Since almost all patients managed operatively in our study had no lymph node metastases on the PSMA PET-CT and no positive lymph nodes were retrieved at surgery, it was not possible to meaningfully assess broader statistical measures of diagnostic performance such as sensitivity and specificity compared to histopathology. Furthermore, in the absence of histopathological correlation in the large number of patients who did not undergo operative management, assessment of PSA remission and long-term outcome data could act as a surrogate gold standard for PET-CT performance. Importantly, the observed instances of BCR would suggest that very small volume and micrometastatic disease remain beyond the detectable threshold of PET-CT despite the histopathological concordance in this study. This makes sense given the intrinsically limited spatial resolution of PET-CT imaging although alternative radiopharmaceuticals, such as newer generation 18F-labelled PSMA compounds, may provide incremental improvements in performance. Determining the clinical significance of the PSMA PET-CT findings requires longer term follow-up.

The role of PSMA PET-CT in the management of high risk PCa patients remains the subject of ongoing research. Our study highlighted a number of contemporaneously identified examples where the individual surgeon’s preference to offer surgical management was significantly altered by unexpected findings on the PSMA PET-CT scan, predominantly up-staging patients who may have avoided unnecessary surgery based on conventional imaging alone. In a recent multicentre prospective Australian study, PSMA PET-CT demonstrated a change in all-modality management intent of 21% in a cohort of both intermediate and high risk primary prostate cancer. The impact in the setting of BCR was significantly greater at 62% [6]. The long-term clinical outcomes and financial impact of these findings could be the subject of future analysis.

Our study was performed in a single centre with experienced nuclear medicine physicians following standardisation of interpretation criteria using the proposals by Fanti et al. [12]. Unlike FDG PET-CT where internal reference standards such as uptake in the blood pool or liver can assist in interpretation, no consensus guidelines for standardised reporting criteria exist in PSMA PET-CT. Reader experience may also impact the diagnostic performance of the study. For example, the poorest sensitivity of primary staging PSMA PET-CT scanning reported in the literature was 33% however, this study that did not involve specialists in reporting PET-CT scans [7].

## Conclusions

Our study found that PSMA PET-CT appears to have a high negative predictive value for local lymph node metastases in patients with high risk primary PCa who undergo RP-PLND compared to histopathological findings. Ongoing follow-up is needed to assess the long-term clinical impact of these findings.

## Data Availability

Please contact the corresponding author regarding any requests for the data used or analysed in this study.

## Abbreviations

68Ga: Gallium-68
BCR: Biochemical Recurrence
EAU: European Association of Urology
PCa: Prostate Cancer
PET-CT: Positron Emission Tomography/Computed Tomography
PLND: Pelvic Lymph Node Dissection
PSA: Prostate Specific Antigen
PSMA: Prostate Specific Membrane Antigen
RP: Radical Prostatectomy
VCAR: Volume Computer Assisted Reading
